# Role of solute carriers in response to anticancer drugs

**DOI:** 10.1186/2052-8426-2-15

**Published:** 2014-05-27

**Authors:** Qing Li, Yan Shu

**Affiliations:** Department of Pharmaceutical Sciences, School of Pharmacy, University of Maryland at Baltimore, Baltimore, Maryland USA; Institute of Clinical Pharmacology, Central South University, Changsha, Hunan 410078 China

**Keywords:** Anticancer drugs, Solute carriers (SLC), Chemosensitivity, Chemoresistance

## Abstract

Membrane transporters play critical roles in moving a variety of anticancer drugs across cancer cell membrane, thereby determining chemotherapy efficacy and/or toxicity. The retention of anticancer drugs in cancer cells is the result of net function of efflux and influx transporters. The ATP-binding cassette (ABC) transporters are mainly the efflux transporters expressing at cancer cells, conferring the chemo-resistance in various malignant tumors, which has been well documented over the past decades. However, the function of influx transporters, in particular the solute carriers (SLC) in cancer cells, has only been recently well recognized to have significant impact on cancer therapy. The SLC transporters not only directly bring anticancer agents into cancer cells but also serve as the uptake mediators of essential nutrients for tumor growth and survival. In this review, we concentrate on the interaction of SLC transporters with anticancer drugs and nutrients, and their impact on chemo-sensitivity or -resistance of cancer cells. The differential expression patterns of SLC transporters between normal and tumor tissues may be well utilized to achieve specific delivery of chemotherapeutic agents.

## Review

**Figure 1:**
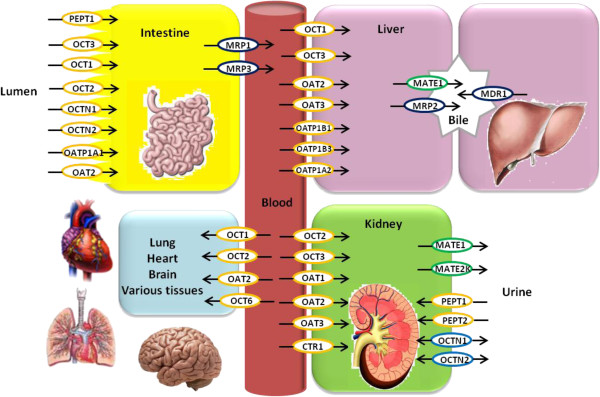
**Schematic model of the transporters in major organs responsible for drug disposition.** The SLC members reviewed here may play an important role in determining the pharmacokinetics of anti-cancer drugs. They may be involved in drug absorption at the intestine, drug uptake into the liver for metabolism, drug elimination in the kidney, and drug distribution in a variety of additional tissues such as heart, lung, and brain. To facilitate understanding of drug transport processes in the organs, important efflux ABC transporters, which are reviewed elsewhere, are also depicted. Influx SLC transporters are shown as yellow open ovals and efflux SLC transporters as green open ovals. Some SLC transporters which mediate bi-directional transport are depicted by blue open ovals. ABC transporters are shown in black ovals. Black solid arrows indicate the direction of drug transport.

Membrane transporters play critical roles in moving a myriad of endogenous and exogenous substances across cellular and organelle membranes, including the major nutrient metabolites sugars, digested peptides, amino acids and nucleosides, rare elements such as hormones and neurotransmitters, and xenobiotics. These transporters are encoded by numerous gene families, accounting for approximately 4% of genes in human genome [1]. Clinically used drugs usually have similar physiochemical structures with certain endogenous substrates. In the last two decades, a variety of membrane transporters have been recognized as drug transporters. These drug transporters are of essential importance in determining drug pharmacokinetics, efficacy and adverse effects. Based on the driving force, drug transporters are characterized by two major superfamilies, the solute carriers (SLC) and the ATP-binding cassette (ABC) transporters which can each be functionally classified into influx and efflux transporters according to the direction of movement of substrates (Figure 1).

As reviewed elsewhere, the ABC transporters, which are typical efflux drug transporters using energy from the hydrolysis of ATP as the driving force to move substrates against the electrochemical gradients outward or into intracellular organelles. The 49 ABC transporters can be classified into 7 subfamilies from A to G subfamily [2–5]. In the past two decades, accumulating evidence has shown that enhanced expression of several ABC transporter genes is associated with reduced cellular accumulation of anticancer drugs and acquired multidrug resistance in many human cancer cells. Those ABC transporters of importance in cancer therapy have been well documented for ABCB1 (or MDR1), ABCC1 (or MRP1), ABCC2 (or MRP2), and ABCG1 (or BCRP) [6–8]. By contrast, the impact of the solute carriers (SLC), which are usually influx or bi-directional transporters, on cancer therapy has not been extensively characterized. In this review, we provide updates on the documented interaction of SLC transporters with anti-cancer drugs.

**Figure 2:**
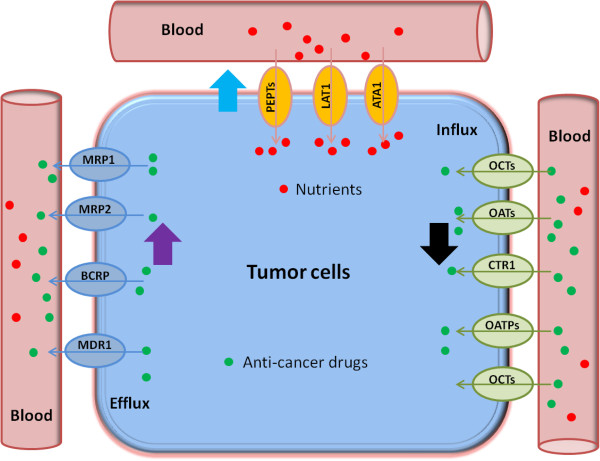
**Schematic model of development of anticancer drug resistance due to altered expression of transporters.** SLC transporters (OCTs, OATs, CTR, and OATPs in green open ovals) may take up anticancer drugs into the cancer cells, while some ABC transporters (MDR1, MRP1, MRP3, and BCRP in blue open ovals) may extrude them. In addition, certain SLC transporters (PEPTs, LATs, and ATA1 in yellow open ovals) mediate the uptake of nutrients (e.g., amino acids and peptides) into cancer cells for their survival. The decreased expression of SLC transporters (black bold arrow) responsible for drug uptake or/and the increased expression of ABC transporter (purple bold arrow) for drug efflux make the cancer cells more resistant to anticancer drugs. The increased expression of SLC transporters (blue bold arrow) responsible for nutrient uptake may also cause more resistant to anticancer drugs as the cancer cells have an advantage of survival from limited nutrient supply.

More than 400 SLC transporter genes have been identified and grouped into 55 families, including ion coupled transporters, exchangers and passive transporters located at the plasma membrane or in intracellular organelles (e.g. mitochondrial or vesicular transporters) [9]. Unlike ABC transporters which driving energy is provided by hydrolysis of ATP, SLC proteins work either by facilitating passive diffusion along the concentration gradient of the substrate or by co-transport and counter-transport against the concentration gradient of another solute. The superfamily of SLCs is responsible for mediating the transport of a wide spectrum of substrates, including different nutrients as well as drugs [10].The SLC transporters expressed in the small intestine, the liver, and the kidney may be of particular importance for the disposition of cancer drugs. Interindividual variation in the activities of these transporters may cause altered pharmacokinetic profiles of anticancer drugs, subsequently leading to variability in the pharmacodynamic effects. The SLC transporters expressed in cancer cells play an important role in cellular uptake of cancer drugs, which may be a determinant step toward anti-cancer efficacy. Indeed, cancer cells are more likely to show substantially different expression profiles of SLC transporters as compared to those of normal cells. Moreover, by mediating the transport of essential nutrient molecules and modulating the electrochemical gradient across the membranes, SLC proteins can function to modify the efficiency of drug diffusion into cells or alter cell survival pathways, consequently influencing chemotherapeutic efficacy (sensitivity or resistance). In certain cases, cancer cells may possess enhanced expression of SLC transporters for certain nutritional requirements and take a growth advantage over normal cells when nutrients become restricted (Figure 2).

**Figure 3:**
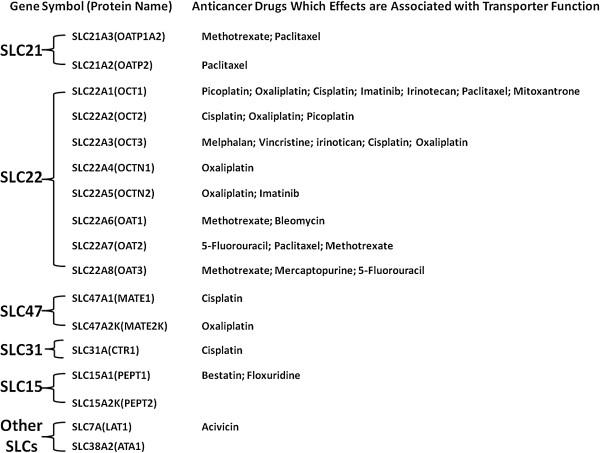
**Classified subfamilies of SLC transporters involved in response to anticancer drugs.** Only the SLC members and the anticancer drugs reviewed in this article are included.

To improve outcomes of cancer therapy, it is necessary to fully characterize the function of SLC drug transporters in the organs critical to the disposition of anti-cancer drugs, such as intestine, liver and kidney. Moreover, by understanding the altered expression of SLC transporters in various cancer cells, we may develop novel therapeutic strategies to treat cancers. For instance, transporter-targeted chemotherapy may be achieved *via* down-regulation of certain essential transporters for cancer cell survival. The major subfamilies of SLC members, which are, to different extents, explored in their association with cancer therapy, include the following: folate transporters (SLC19A1 and SLC 46A1), which are particularly important for antifolate chemotherapy of cancer and reviewed elsewhere [11–13]; organic cation transporters (OCT) (SLC22A1-3); organic anion transporters (OAT) (SLC22A6-8); organic cation/carnitine transporters (SLC22A4-5); organic anion transporters polypeptides (OATPs) (SLCO); copper transporters (SLC31A); multidrug and toxin extrusion proteins (MATEs) (SLC47A), which intriguingly function as efflux transporters in certain tissues; oligopeptide transporters (SLC15A1/2); and amino acid transporters (SLC7A and SLC3A) (Figure 3).

### Organic cation transporters (OCT)

Organic cation transporters consist of three isoforms (OCT1/SLC22A1, OCT2/SLC22A2, OCT3/SLC22A3), which mediate the transport of various organic cations, weak bases, and some neural compounds across plasma membranes [14, 15]. These transporters are facilitative diffusion systems, and the driving force is provided by the electrochemical gradient of the transported compounds [16].

### OCT1

Human OCT1 (SLC22A1) is predominantly expressed in the liver where it is located in the sinusoidal membrane of the hepatocytes [17]. In rat, mouse, and rabbit, besides high expression in the liver, strong expression was also detected in the kidney and it was localized at the basolateral membranes of S1 and S2 segments of proximal tubules [18]. Human OCT1 is expressed to a much lesser extent in various additional organs including kidney, small intestine, lung, heart, skeletal muscle, brain, placenta, mammary gland, adrenal gland, eye, adipose tissue, and immune cells [18–23], and differentially expresses in various tumors [18, 24, 25].

OCT1 has been showed to be expressed in colon cancer and polyps [26]. Specifically, it has been reported that OCT1 mRNA level is increased in human colon cancer cell lines and patient-derived colorectal tumor samples [27]. Platinum-based drugs, including cisplatin and oxaliplatin, are efficient to induce DNA damage by forming DNA adducts and subsequently cause apoptosis in colon cancer cells. Cisplatin has shown a high specificity and affinity for OCT1 with an IC_50_ (half maximal inhibitory concentration) value of 8.1 uM. However, Zhang *et al*. reported that human OCT1 markedly increased oxaliplatin, but not cisplatin, accumulation and cytotoxicity in the stable cells expressing OCT1 transporter and oxaliplatin was an excellent substrate of OCT1 [28]. The cytotoxicity by oxaliplatin was also much greater than that of cisplatin in the colon cancer cells. By using an OCT inhibitor, cimetidine, they demonstrated that the function of OCTs such as OCT1 and OCT2 is a major determinant of the anticancer activity of oxaliplatin, which may be causative of its specificity of anti-colon cancer. Picoplatin, a third-generation platinum agent, is efficacious against lung cancers that become refractory to other platinum-based treatment. More *et al*. reported that the tumor size of OCT1-expressing xenografts in mice was significantly reduced by picoplatin treatment as compared to control xenografts, which suggested that OCT1 could enhance the antitumor efficacy of picoplatin as well [25].

OCT1 has also been demonstrated to be significantly expressed in chronic myeloid leukemia (CML) cell lines and primary CML cells [29]. Imatinib, a potent tyrosine kinase inhibitor, is used to treat CML. OCT1 has been reported to mediate the transport of imatinib mesylate across cellular membrane. Considerable evidence has revealed that OCT1 expression level in leukemic cells is associated with the therapeutic outcome in CML [30–35]. It has been reported that OCT1 activity may be a key determinant of molecular response to imatinib [36]. By using a potent inhibitor such as prazosin, the activity of OCT1-mediated imatinib transport can be determined *in vitro* in the isolated peripheral blood leukocytes from CML patients. This measurement of OCT1 function may be useful to individualize dosage regimens for patients with CML in order to obtain an optimal outcome in the long-term imatinib-treated patients [36].

In addition, the antineoplastic agents irinotecan, mitoxantrone, and paclitaxel were found to inhibit the uptake of the organic cation ^3^H-1-methyl-4-pyridinium iodide into Chinese hamster ovary cells that are overexpressed with human OCT1, with K_i_ values of 1.7, 85, and 50 μM, respectively [24]. The OCT1-transfected cells also exhibited significantly more susceptible to the cytotoxicity of irinotecan and paclitaxel when compared with mock cells, suggesting that OCT1 may contribute to accumulation of the selected antineoplastic drugs in cancer cells. The expression of OCTs in various tumors should be further investigated as it may serve as a biomarker for selecting specific antineoplastic agents to tailor cancer therapy for individual patients.

### OCT2

The cloning of Slc22a2 encoding Oct2 from rat was reported in 1996 [37]. OCT2 orthologs were later cloned from other species including humans [14, 38]. Human OCT2 (SLC22A2) is mainly expressed at the basolateral membrane of renal proximal tubules in the kidney [14], and its expression is also detectable in small intestine, lung, placenta, thymus neurons, choroid plexus, inner ear, and respiratory mucosa [39, 14].

OCT2 mediates the first step of many cationic drugs in renal excretion *via* their uptake at the basolateral membrane of proximal tubule cells. As for anticancer drugs, cisplatin has been demonstrated to be a substrate of OCT2, evidenced by significantly higher uptake of cisplatin in OCT2 overexpressing cells compared with that in mock cells [39]. Abundant evidence indicates that cisplatin accumulates in the proximal tubules and causes nephrotoxicity. Human OCT2 transporter has been reported to mediate the transport of cisplatin across proximal tubules membrane from circulation [40], and is critical to the nephrotoxicity of cisplatin. A non-synonymous single-nucleotide polymorphism (SNP, *rs316019*) in the OCT2 gene has been demonstrated to be associated with decreased nephrotoxicity of cisplatin in patients [40]. Moreover, the mice lacking of Oct2 function have reduced urinary excretion of cisplatin and less cisplatin-induced nephrotocixity [40]. In addition, the wild-type mice treated with cisplatin plus the OCT inhibitor cimetidine have a reduced nephrotoxicity similar to that seen in Oct1/2-/- double knockout mice treated with cisplatin alone [41]. It seems that a reduced uptake by transporters such as OCT2 at the basolateral membrane in the proximal tubules is likely to protect kidney from cisplatin-induced nephrotoxicity. Oxaliplatin has also been considered as a substrate of OCT2. However, it does not induce severe nephrotoxic effects similar to cisplatin [42]. This may be explained by another protein transporting organic cations, the kidney isoform of MATE2 (MATE2K). MATE2K (SLC47A2) is located at the apical membrane of proximal tubules and can specifically remove oxaliplatin, not cisplatin, from the proximal tubules [43–45]. In addition, the third-generation platinum drug, picoplatin, has also been shown to significantly enhance cytotoxicity in the presence of OCT2 expression with increased DNA adduct formation [25]. These studies underscore the importance of OCT2 function in renal disposition and possible tumor accumulation of platinum compounds in platinum-based chemotherapy.

### OCT3

The SLC22A3 gene encoding OCT3 was independently cloned as the extraneuronal monoamine transporter by different groups [46, 47]. Unlike human OCT1 and OCT2 with high expression in limited tissues, human OCT3 is ubiquitously expressed [15, 18, 48] with relatively high expression in the heart, skeletal muscle, brain, liver, and kidney [14, 47, 49]. Specifically, human OCT3 is located to the basolateral membrane of placental epithelium [50], the sinusoidal membrane of hepatocytes [17], and the luminal membrane of bronchial epithelial cells [18].

Human OCT3 has differential expression between normal and cancerous tissues. An upregulated OCT3 expression was observed in the cancerous tissues of colon rectum and stomach as compared to the normal tissues, however, significantly decreased expression was found in several other cancerous tissues, including uterus, breast, ovary and lung cancers [51]. It has been reported that oxaliplatin is a substrate of OCT3, in contrast to carboplatin, which are not substrate of OCT3 [43, 44]. OCT3 moderately transports oxaliplatin and can mediate oxaliplatin-induced cytotoxicity in cell cultures [51]. Yokoo *et al*. reported that the effect of oxaliplatin against colorectal cancer was superior to that of cisplatin because OCT3 was highly expressed when compared with other organic cation transporters [51]. Recent studies have showed that other cytostatics, such as melphalan, irinotecan, and vincristine [52], may interact with OCT3 as well. Irinotecan, which is well used to treat colon carcinoma, exhibits a high affinity for OCT3, and its increased cytotoxicity in kidney carcinoma cell lines was associated with OCT3 expression. These results suggested that human OCT3 expression may be further evaluated as a potential biomarker for the efficacy of cancer chemotherapy.

### Organic anion transporters (OAT)

Organic anion transporters (OATs), also members of the solute carrier family 22, play a key role in renal excretion of water-soluble, negatively charged organic compounds [53]. Renal OATs are expressed in the baselateral membranes of proximal tubular cells, the major site for secreting organic anions in the kidney. OATs are also expressed in other tissues, including liver, placenta, nasal epithelium, and blood-brain barrier. In humans, OAT subfamily consists of OAT1–4, OAT7, OAT10, and URAT1 (SLC22A16). Several classes of drugs interact with human OAT1-3, including ACE inhibitors, angiotensin II receptor antagonists, diuretics, HMG CoA reductase inhibitors, β-lactam antibiotics, antiviral drugs, uricosuric drugs, and antineoplastic drugs. In this review, we focus on OAT1-3, whose role in drug disposition and cancer therapy has been investigated relatively well.

### OAT1

The organic anion transporter 1, OAT1 (SLC22A6), was the first OAT member which was cloned from rat [54], mouse [55], and later human [56, 57]. Human OAT1 was localized to the basolateral membrane of renal proximal tubule cells by immunocytochemistry and was found along the whole proximal tubule [57–59]. This expression pattern is consistent with the role of OAT1 in the uptake of anionic drugs from the circulation into proximal tubule cells. OAT1 has also been detected in the brain, specifically in the choroid plexus [60].

Human OAT1 has been reported to be responsible for renal tubular secretion of methotrexate which is an antifolate used in the treatment of malignancies [61]. Methotrexate is also transported by rat and mouse Oat1 with a moderate to high affinity [62, 63]. Clinical interaction between methotrexate and other drugs such as loxoprofen has been well documented. Interestingly, loxoprofen and its trans-OH metabolite, the major active metabolite, markedly inhibit the transport of methotrexate by OAT1. In clinical chemotherapy, high doses of methotrexate are frequently used, and methotrexate-induced severe nephrotoxicity has been well documented. OAT-mediated uptake of methotrexate into tubule cells may significantly contribute to its nephrotoxicity. Reports are also available suggesting that the cytotoxicity and anticancer effects of several other antineoplastic agents, including 6-mercaptopurine, azathioprin, cisplatin, imatinib, cytarabine, vinblastine, vincristine, hydrocortisone, and mitoxantrone, may be associated with OAT1 expression [64, 65].

### OAT2

Liver and kidney have the highest expression level of OAT2, and relatively low expression level of OAT2 was found in pancreas, small intestine, lung, brain, spinal cord, and heart. [66–69]. However, the expression of OAT2 mRNA is much lower than those of OAT1 and OAT3 mRNAs In human kidneys. [67]. OAT2 is located in the sinusoidal membrane of hepatocytes [70] and the basolateral membrane of proximal tubule cells [71].

As a major hepatic transporter, OAT2 plays an important role in hepatic drug uptake, which may be a determinant of metabolism for various anticancer drugs. 5-fluorouracil, which is used in colorectal and pancreatic cancer therapy, is a verified substrate of OAT2 [72]. In the same study, Kobayashi *et al*. also reported that paclitaxel was an OAT2 substrate [72]. Tanino *et al*. later showed a concentration-dependent uptake of paclitaxel in rat hepatocytes [73]. However, indomethacin, a representative inhibitor of Oat2, did not significantly inhibited the uptake of paclitaxel, which suggested that Oat2 was not a significant transporter for the hepatic uptake of paclitaxel, at least in rat hepatocytes [73].

### OAT3

OAT3 (SLC22A8) has been cloned from human, monkey, pig, rabbit, rat, and mouse. In these species, OAT3 mRNA is detected in a variety of tissues or organs including kidney, liver, brain, skeletal muscle, and adrenal glands [65, 74]. In all tested species, the kidney has the highest mRNA expression of OAT3 among all organs [21, 74]. The transporter protein is located at the basolateral membrane of proximal tubule cells in the kidney [38, 75].

Methotrexate (MTX) has a 30-fold stronger affinity to OAT3 with the K_m_ value of 17.2 μM as compared with OAT1 (K_m_: 724 μM) [61]. Thus, human OAT3 could be mainly responsible for the renal MTX excretion and MTX-induced nephrotoxicity during cancer therapy. Among interactions between anticancer drugs and OAT3, 6-thioguanine was found to inhibit rat Oat3 (IC_50_ 172 μM), 6-mercaptopurine could be transported by both rat and mouse Oat3 (K_m_: 50.5 μM and 4 μM, respectively), and 5-fluorouracil was substrate of mouse Oat3 (K_m_: 0.054 μM) [76, 64]. In addition, rat Oat3 (K_m_: 21.9 μM) and human OAT3 (K_m_: 56.5 μM) translocated topotecan with a moderate affinity [77].

### Organic cation/carnitine transporters (OCTN1-2 and OCT6)

Organic cation/carnitine transporters comprise the transporters OCTN1, OCTN2 and OCT6, encoded by the genes SLC22A4, SLC22A5 and SLC22A16, respectively [18]. The three transporter genes have a broad expression pattern in normal tissues. Some human tumor-derived cell lines, including lung carcinoma A549, colorectal carcinoma SW480, and cervix carcinoma Hela S3 cells, have been detected to express high levels of OCTN1 and OCTN2 transcripts [78, 79]. Human OCT6 expression has been determined from testis, hematopoietic cells, and leukemia lines HL-60 and MOLT4 [80–82].

Human OCTN1 mediates uniport of organic cations such as the antioxidant ergothioneine as well as H^+^/organic cation antiport, whereas human OCTN2 and OCT6 are Na^+^/carnitine cotransporters as well as organic cation uniporters [18]. Recently, by conducting uptake studies in the HEK293 cells over-expressing rat Octn1, rat Octn2, human OCTN1, and human OCTN2, Jong *et al*. reported that the transport of oxaliplatin across cellular membrane could be mediated by OCTN1 and OCTN2. Both the uptake and cytotoxicity of oxaliplatin were well inhibited by ergothioneine and L-carnitine. Importantly, they provided evidence supporting that the transport of oxaliplatin mediated by OCTN1 appeared to mainly contribute to its neuronal accumulation and treatment-limiting neurotoxicity [83].

OCTN2 has been recently suggested by Hu *et al*. as an efficient and capable transporter for imatinib at a concentration (e.g., 0.2 μmol/L) [84] that is readily achievable in human patients [85]. Furthermore, among the patients of gastrointestinal stromal tumors (GIST) who received imatinib treatment, the time to progression was recently shown to be significantly improved in the carriers of the C allele of an OCTN1 polymorphism (*rs1050152*) as well as in carriers of minor alleles of two OCTN2 polymorphisms (*rs2631367* and *rs2631372*), suggesting the activities of OCTN1 and OCTN2 as a predictor of chemotherapeutic efficacy of imatinib [86].

Doxorubicin is a widely used anticancer drug for hematological malignancies, particularly for acute lymphocytic leukemia (ALL) and acute myeloid leukemia (AML). OCT6 has been found by Okabe *et al*. to mediate the uptake of doxorubicin in leukemic cells and the stable leukemic Jurkat cells with over-expression of *OCT6* gene became more sensitive to doxorubicin. In the same report, OCT6 expression was detected in the primary blood cells collected from the patients with acute leukemia [81]. Bleomycin is widely used in combination with other antineoplastic agents to effectively treat lymphomas, testicular carcinomas, and squamous cell carcinomas of the cervix, head, and neck. OCT6 has also been found to be involved in the uptake of bleomycin-A5 as well as polyamines. Human testicular cancer cells overexpressing OCT6 were extremely sensitive to bleomycin-A5 and the siRNA targeted OCT6 induced significant resistance to bleomycin-A5-dependent genotoxicity. The knowledge of OCT6 function remains relatively limited. These recent findings suggest that characterizing OCT6 expression in human cancer cells may be valuable for us to improve current chemotherapy and explore novel cancer therapeutic strategies [87].

### Organic anion transporting polypeptides (OATP, SLCO)

There are 11 known human OATPs which have been divided into six subfamilies based on their amino acid sequence similarities [88–91]. OATPs expression can be either tissue-specific or ubiquitous in multiple tissues throughout the body. Among the OATP family members, OATP1A2, OATP1B1, OATP1B3 and OATP2B1 have broad substrate specificity and accept a number of therapeutic agents. Latest studies have demonstrated that OATP expression is differently regulated in certain cancer tissues as compared to normal tissues. The OATP1 subfamily is the most characterized among the OATPs. It should be pointed out that very different subfamily members of SLCO/Slco have been evolved between humans and rodents. This is particularly true within the OATP1 family as there are no clear orthologs between individual human and mouse OATP1s. Humans have only one OATP1A transporter (OATP1A2), but mice have at least 4 (Oatp1a1, Oatp1a4, Oatp1a5, and Oatp1a6) [89, 92]. In addition, while humans have 2 OATP1B transporters (OATP1B1 and OATP1B3), mice only have Oatp1b2 [92]. In this section, we focus on the interaction of OATP1 with anticancer drugs.

### OATP1B1

OATP1B1 (SLCO1B1) is highly expressed in human liver [93–95]. Further, the transporter gene profiling assay in NCI-60 cancer cell lines showed relatively high expression of OATP1B1 in the cells derived from lung cancers, such as A549 and EKVX cells, and colon cancers, such as HCT-15 and KM12 cells [96]. In addition, the mRNA level of OATP1B1 was found to be higher in colon polyps and cancer tissues as compared with normal colon tissues [26].

OATP1B1 activity may be important in hepatic disposition of anticancer drugs. Nozawa *et al*. firstly reported that OATP1B1 is responsible for hepatic uptake of SN-38, the major active metabolite of irinotecan and that the genetic polymorphisms of OATP1B1 may contribute to the well-known individual variation in the disposition of irinotecan [97]. Later, in a case report, severe irinotecan toxicity was observed in a 66-year-old Japanese with dysfunctional alleles of OATP1B1 [98]. The toxicity is possibly due to a decreased uptake of active metabolite (SN-38) of irinotecan into the liver and subsequently reduced hepatic metabolism. It has been demonstrated that in OATP1B1 transgenic mice, the hepatic accumulation of MTX was significantly higher (approximately 2-fold) compared with wild-type mice after MTX treatment, resulting in 2- to 4-fold higher liver-plasma ratios of MTX. The findings suggest a marked and possibly rate-limiting role for human OATP1B1 in MTX elimination *in vivo*[99]. In addition, polymorphisms in the OATP1B1 gene were found to be associated with the disposition and therapeutic outcomes of flavopiridol and atrasentan [100, 101].

### OATP1B3

OATP1B3 (SLCO1B3), normally and specifically expressed in liver, has been found in different cancer tissues [102]. Specifically, OATP1B3 is upregulated in gastrointestinal cancer cell lines, pancreatic cancer cell lines, and gallbladder cancer cell lines. OATP1B3 expression was dramatically higher in colorectal adenocarcinoma tissues and in prostate cancer tissues as compared with their corresponding normal tissues [103–105].

Hu *et al*. found a significantly higher uptake rate for [^3^H] imatinib in HEK293 cells transfected with human OATP1B3 gene and in *Xenopus laevis* oocytes injected with OATP1B3 cRNA [84]. Methotrexate has also been demonstrated as an OATIP1B3 substrate as OATP1B3 transports methotrexate in a saturable and dose-dependent manner. The introduction of the *OATP1B3* gene into mammalian cells potentiates their sensitivity to methotrexate [106]. In *Xenopus laevis* oocytes injected with OATP1B3 cRNA, the uptake of docetaxel and paclitaxel were 2.2-fold and 3.3-fold higher, respectively, than that of water-injected control oocytes [107, 108], suggesting both drugs as OATP1B3 substrates. However, in screening interaction between OATP1B3 and a variety of compounds, although docetaxel, paclitaxel, and three other antineoplastic agents, actinomycin D, mitoxantrone, and SN-38 exhibited potent inhibitory effects on OATP1B3-mediated transport of CDCA-NBD (chenodeoxycholyl-(Nepsilon-NBD)-lysine, a fluorescent substrate of OATP1B3)) [109], only SN-38 was further determined as a novel substrate for OATP1B3 [110]. Overall, OATP1B3 may be a clinically relevant transporter responsible for hepatic disposition and the chemotherapeutic response in cancer tissues of certain anticancer drugs.

### OATP1A2

Among normal tissues, OATP1A2 (SLCO1A2) is expressed in the intestinal epithelium [111], the renal epithelium, and highly in brain capillary endothelial cells. [112] Altered OATP1A2 expression has been detected in glioma, colon polyps and cancers, and breast cancers as compared to normal tissues. In addition, OATP1A2 mRNA were found both in bone metastases from primary kidney cancer and in the malignant osteosarcoma cell lines HOS and MG-63 [113].

Paclitaxel has been characterized as an OATP1A2 substrate. When compared to wild-type mice, the transgenic mice overexpressing human OATP1A2 had a remarkable increased hepatic uptake of paclitaxel [114]. Furthermore, the systemic exposure of paclitaxel after an intravenous dose (10 mg/kg) was increased by greater than 2-fold in Slco1a/1b (-/-) mice compared with wild-type mice, whereas its hepatic uptake was reduced by about 2-fold [115]. There is *in vivo* evidence supporting methotrexate (MTX) as a substrate of OATP1A2 as well. When compared with wild-type mice, Slco1a/1b (-/-) mice exhibited a 3.4-fold increase in plasma and 30-fold decrease in hepatic levels of MTX after received a high dose of 500 mg/kg, suggesting an overall role of OATP function in MTX disposition [115]. Moreover, humanized OATP1A2 transgenic mice showed significant rescue of the increased plasma levels and decreased liver and small intestinal accumulation of MTX that were observed in Slco1a/1b (-/-) mice [114], confirmed MTX as an OATP1A2 substrate. OATP1A2 has been extensively detected in primary and metastatic liver cancers. As the transporter could be a critical mediator of drug uptake in the liver, OATP1A2 may be further exploited for the delivery of chemotherapeutic agents to treat liver cancers [116].

### Copper transporters (CTR, SLC31A)

Copper is a vital mineral for humans as demonstrated by serious health concerns associated with its deficiency or excess accumulation. Copper transporter 1 (CTR1) is the major high-affinity copper uptake transporter in mammals [117]. CTR1, encoded by the *SLC31A1* gene, is localized in the plasma membrane and intracellular membranes [118, 119]. CTR1 protein is ubiquitously expressed in many tissues such as hepatocytes, α cells of the pancreatic islets, enteroendocrine cells of the gastric mucosa and bronchioles, c cells of the thyroid, and a subsets of cells in the anterior pituitary [120]. It is also observed to be expressed in both normal colonic epithelium and colon carcinomas [120]. In addition, strong expression has been found in a few cases of carcinoid tumors, Ewing’s sarcoma, undifferentiated carcinomas, and enteroendocrine cells [120].

Abundant evidence has been consistently showed that CTR1 is an important determinant of cellular accumulation and toxicity of platinum-based anti-cancer drugs, such as cisplatin [121–123]. Stable overexpression of human CTR1 significantly increased the uptake of cisplatin, carboplatin and oxaliplatin in human small cell lung cancer cell lines and rendered the cells more sensitive to the cytoxicity of these compounds [124, 125]. In contrast, small interfering RNA (siRNA)-mediated knockdown of CTR1 was shown to be able to reduce both cellular platinum accumulation and cytotoxicity of cisplatin in human embryonic kidney 293 cells [126]. In addition, mouse embryonic fibroblasts (MEF) of heterozygous (+/-) and homozygous (-/-) *Ctr1* gene deletion accumulated 35% and 70% less platinum and exhibited 4- and 8-fold more resistant to cisplatin cytotoxicity after a 2 hour cisplatin exposure, respectively, than the wild-type MEF cells [121]. Taken together, these studies have provided strong evidence in support of CTR1 role in mediating the uptake of cisplatin into normal and tumor cells. Further clinical studies are needed to clarify how important the transporter function is in determining the anticancer efficacy and the toxic side effects of platinum-based treatment in patients.

### Multidrug and toxin extrusion (MATE) proteins (SLC47A)

Multidrug and toxin extrusion (MATE) proteins were first found in bacteria in 1998 [127]. In 2005, the human ortholog, MATE1, was first cloned and characterized as an efflux transporter mediating the excretion of organic cations from the kidney [128]. MATE2 and MATE2-K were identified shortly thereafter. The driving force for MATE is provided by the oppositely directed proton gradient [128]. MATE proteins exist in various living organisms, including prokaryotes, plants and mammals, and they are responsible for the transport of various endo-/exogenous substrates. In humans, MATE1 is highly expressed in the kidney and liver, which is localized at the apical membrane of proximal tubules and hepatocytes. Human MATE1 is also detectable in other tissues, including adrenal gland, skeletal muscle, testis, and first trimester placenta [129, 130]. MATE2 and MATE2-K are mainly expressed in the kidney [131].

Otsuka *et al*. reported that MATE1 mediates the excretion of cisplatin in the kidney [128]. It has been lately reported by Nakamura *et al*. that cisplatine significantly increased the levels of plasma creatinine and blood urea nitrogen (BUN), two major biomarkers for renal injury, in *Mate1*-deficient (*Mate1*-/-) mice when compared with wild-type mice [132]. Further, the levels of creatinine and BUN in the mice treated with cisplatin were significantly enhanced by pyrimethamine, a potent MATE inhibitor [132]. Li *et al*. later reported that much severer nephrotoxicity of cisplatin was observed in *Mate1*-/- mice than in wild-type mice [133]. Thus, reduced function of MATEs, which serves as efflux transporters for cisplatin elimination in the kidney, may be responsible for cisplatin-induced nephrotoxicity. In addition to cisplatin as a substrate of MATE1, oxaliplatin was reported to be transported by rat Mate1 as well as human MATE1 and MATE2-K [134].

### Oligopeptide transporters (PEPT1/2, SLC15A1/2)

The oligopeptide transporters (PEPTs, SLC15A) serve as integral membrane proteins for mediating the cellular uptake of di- and tripeptides into cells. Their driving force comes from an inwardly directed H^+^ gradient across the membrane [135]. PEPT1 and PEPT2 are two major PEPTs that have been cloned. Physiologically, PEPTs are localized at brush-border membranes of intestinal and renal epithelial cells, and play important roles in protein absorption and the conservation of peptide-bound amino nitrogen. Peptide-like drugs with structural similarities to di- and tripeptides are also transported by PEPTs [136]. PEPT1, a high-capacity but low affinity transporter, mainly expressed in the small intestine, whereas PEPT2, a high-affinity but low-capacity transporter, broadly expressed in a variety of tissues [135].

PepT1 and PepT2 were interestingly found to be expressed in fibroblast-derived tumor cells but not in normal fibroblasts [137]. High levels of PEPT 1 protein were also detected in two human pancreatic cancer cell lines, AsPc-1 and Capan-2 [138]. In addition, PEPT1 mRNA was increased 2.3-fold in colon cancer tissues as compared to normal tissues [139]. Many peptide-mimetic agents are the substrates of PEPTs. The higher expression of PEPTs in cancer cells may serve as the basis of a novel strategy for specific delivery of oligopeptide-mimetic anticancer drugs into tumors.

Bestatin, a potent aminopeptidase inhibitor and a known substrate of PEPT1, suppressed the growth of tumor (Hela cells) xenografts overexpressing PEPT1 by 4-week consecutive oral administration [140]. The high expression of PEPTs in various cancer tissues has been attributed to increased nutrients demand by fast tumor growth, and thus inhibition of the activity of PEPTs might be also a novel targeting strategy to delay or stop tumor growth. Mitsuoka *et al*. found that a newly synthesized dipeptide, 4-(4-methoxyphenyl)-L-phenylalanyl sarcosine which was an inhibitor of PEPT1, resulted in nearly complete suppression of the xenograft growth of human pancreatic cancer AsPC-1 cells that highly expressed PEPT1 [141].

Floxuridine, a clinically proven anticancer drug, is commonly used for the treatment of metastases from colon carcinomas and hepatocellular carcinoma [142]. In order to improve its selectivity and to reduce undesirable toxic effects, a series of prodrugs of floxuridine has been developed [143, 144]. These amino acid ester prodrugs have been shown to target the PEPT1 transporter [145]. MDCK cells stably transfected with the human PEPT1 (MDCK/hPEPT1) demonstrated enhanced cell growth inhibition in the presence of these prodrugs [146]. This prodrug strategy to modify nucleoside drugs seems to have great potential to improve their tumor selectivity and drug efficacy.

### Amino acid transporters

The transporter-mediated entry of amino acids into cells is necessary for various cellular functions including protein synthesis, energy metabolism, glutathione synthesis, and others. Moreover, amino acids transporters play important roles in conferring not only drug sensitivity by mediating the uptake of amino acid analog drugs), but also drug resistance by promoting the uptake of essential amino acids for tumor growth and survival. There are 6 major families of amino acid transporters in the solute carrier (SLC) gene superfamily (SLC1, SLC6, SLC7, SLC36, SLC38, and SLC43 families) and the orphan SLC16 monocarboxylate transporter which transports aromatic amino acids [147].

Accumulating evidence in the past two decades has indicated that amino acid availability controls cellular physiology by altered gene expression levels and signal transduction pathways in cancer cells. For example, the mammalian target of rapamycin (mTOR) is a serine/threonine kinase that regulates fundamental biological processes and plays critical roles in cell growth regulation and tumorigenesis [148, 149]. The activation of mTOR and subsequent regulation of mTORC1 is regulated by the uptake of amino acids such as L-glutamine which has been interestingly shown to be transported by the bidirectional transporters SLC1A5 and SLC7A5 (LAT1) [150]. Regulation of mTOR signaling pathway by modulating the activity of these amino acid transporters could be an attractive strategy to control tumor cell survival and progression.

Amino acid transporter system A1 (ATA1), encoded by *SLC38A2*, mediates the transport of most small neutral amino acids, including alanine, serine, and glutamine. The mRNA level of ATA1 was markedly induced in human hepatoma cancer cell lines and in patient-derived hepatocellular cancer tissues as well. Enhanced expression of ATA1 is also positively related to the progression of cholangiocarcinoma. Furthermore, silencing ATA1 mRNA expression decreased the viability of HepG2 cells, suggesting that ATA1 is likely essential to tumor survival [151]. These studies have indicated the prognostic significance of ATA1 in cancer development and progression and provided rationales to target ATA1 for cancer therapy.

L-type amino acid transporter 1 (LAT1), encoded by *SLC7A5*, is responsible for the transport of large neutral, aromatic or branched amino acids from extracellular fluids into the cells. Acivicin is an antineoplastic antibiotic that targets glutamine-dependent amidotransferases in the biosynthesis of purines and pyrimidines [152]. The IC_50_ values for acivicin to inhibit the gabapentin (a LAT1 substrate) accumulation in the stable HEK-LAT1 cells ranged from 7.9 μM to 340 μM [153]. The experiments of trans-stimulation and cell-proliferation have demonstrated that acivicin is likely to be a substrate for LAT1, suggesting that LAT1 may be targeted for acivicin delivery into tumor cells [153]. Petel *et al*. have recently demonstrated that LAT1 is functionally active in prostate cancer cells (PC-3). Hence, LAT1 transporter may be used as a target for improving the availability of poorly permeable but highly potent anticancer drugs at least in prostate cancer cells [154].

## Conclusions

It is critical to target drugs to tumor cells in order to improve the clinical efficacy and avoid the adverse effects of anticancer drugs. The efficacy of chemotherapy may be largely dependent on the relative activity of transporters in normal and cancer tissues. In addition to already extensively investigated efflux transporters, multiple types of membrane influx transporters, in particular the SLC superfamily members play very important roles in conferring sensitivity and resistance to anticancer agents. These SLC transporters not only directly bring anticancer agents into cancer cells but also serve as the uptake mediators of essential nutrients for tumor growth and survival. The differential expression patterns of SLC transporters between normal and tumor tissues may be well utilized to achieve specific delivery of chemotherapeutic agents. The transporters may be also directly targeted in development of anticancer drugs to increase chemosensitivity, for example, *via* limiting nutrient supply to cancer cells and regulating their apoptosis and electrochemical gradients. The SLC transporters expressed in the intestine, liver and kidney are of particular importance as their activity may be critical to systemic exposure and disposition of various anticancer agents, serving as a common basis or determinant for drug-drug interaction, pharmacological effects, and side effects. The function of SLC transporters in anticancer drug disposition and action has been increasingly recognized. However, the major biological implication and pathophysiological function of these membrane proteins are far from clear and under extensive exploration. With advanced knowledge of SLC transporters, their role in the development, optimization, and personalization of anticancer medicine will be further underscored and merited.

## References

[1] Venter JC, Adams MD, Myers EW, Li PW, Mural RJ, Sutton GG, Smith HO, Yandell M, Evans CA, Holt RA, Gocayne JD, Amanatides P, Ballew RM, Huson DH, Wortman JR, Zhang Q, Kodira CD, Zheng XH, Chen L, Skupski M, Subramanian G, Thomas PD, Zhang J, Gabor Miklos GL, Nelson C, Broder S, Clark AG, Nadeau J, McKusick VA, Zinder N (2001). The sequence of the human genome. Science.

[2] Dean M, Rzhetsky A, Allikmets R (2001). The human ATP-binding cassette (ABC) transporter superfamily. Genome Res.

[3] Ross DD, Doyle LA (2004). Mining our ABCs: pharmacogenomic approach for evaluating transporter function in cancer drug resistance. Cancer Cell.

[4] Choi YH, Yu AM (2013). ABC Transporters in Multidrug Resistance and Pharmacokinetics, and Strategies for Drug Development. Curr Pharm Des.

[5] Sohma Y (2013). [ABC transporter superfamily]. Nihon Yakurigaku Zasshi.

[6] Deeley RG, Westlake C, Cole SP (2006). Transmembrane transport of endo- and xenobiotics by mammalian ATP-binding cassette multidrug resistance proteins. Physiol Rev.

[7] Gottesman MM, Ling V (2006). The molecular basis of multidrug resistance in cancer: the early years of P-glycoprotein research. FEBS Lett.

[8] Ross DD, Nakanishi T (2010). Impact of breast cancer resistance protein on cancer treatment outcomes. Methods Mol Biol.

[9] He L, Vasiliou K, Nebert DW (2009). Analysis and update of the human solute carrier (SLC) gene superfamily. Hum Genomics.

[10] Rask-Andersen M, Masuram S, Fredriksson R, Schioth HB (2013). Solute carriers as drug targets: current use, clinical trials and prospective. Mol Aspects Med.

[11] Matherly LH, Wilson MR, Hou Z (2014). The Major Facilitative Folate Transporters SLC19A1 and SLC46A1: Biology and Role in Antifolate Chemotherapy of Cancer. Drug Metab Dispos.

[12] Desmoulin SK, Hou Z, Gangjee A, Matherly LH (2012). The human proton-coupled folate transporter: Biology and therapeutic applications to cancer. Cancer Biol Ther.

[13] Trippett TM, Bertino JR (1999). Therapeutic strategies targeting proteins that regulate folate and reduced folate transport. J Chemother.

[14] Koepsell H, Schmitt BM, Gorboulev V (2003). Organic cation transporters. Rev Physiol Biochem Pharmacol.

[15] Nies AT, Schwab M (2010). Organic cation transporter pharmacogenomics and drug-drug interaction. Expert Rev Clin Pharmacol.

[16] Koepsell H (2011). Substrate recognition and translocation by polyspecific organic cation transporters. Biol Chem.

[17] Nies AT, Koepsell H, Winter S, Burk O, Klein K, Kerb R, Zanger UM, Keppler D, Schwab M, Schaeffeler E (2009). Expression of organic cation transporters OCT1 (SLC22A1) and OCT3 (SLC22A3) is affected by genetic factors and cholestasis in human liver. Hepatology.

[18] Koepsell H, Lips K, Volk C (2007). Polyspecific organic cation transporters: structure, function, physiological roles, and biopharmaceutical implications. Pharm Res.

[19] Gilchrist SE, Alcorn J (2010). Lactation stage-dependent expression of transporters in rat whole mammary gland and primary mammary epithelial organoids. Fundam Clin Pharmacol.

[20] Minuesa G, Purcet S, Erkizia I, Molina-Arcas M, Bofill M, Izquierdo-Useros N, Casado FJ, Clotet B, Pastor-Anglada M, Martinez-Picado J (2008). Expression and functionality of anti-human immunodeficiency virus and anticancer drug uptake transporters in immune cells. J Pharmacol Exp Ther.

[21] Nishimura M, Naito S (2005). Tissue-specific mRNA expression profiles of human ATP-binding cassette and solute carrier transporter superfamilies. Drug Metab Pharmacokinet.

[22] Zhang T, Xiang CD, Gale D, Carreiro S, Wu EY, Zhang EY (2008). Drug transporter and cytochrome P450 mRNA expression in human ocular barriers: implications for ocular drug disposition. Drug Metab Dispos.

[23] Koepsell H (2013). The SLC22 family with transporters of organic cations, anions and zwitterions. Mol Aspects Med.

[24] Gupta S, Wulf G, Henjakovic M, Koepsell H, Burckhardt G, Hagos Y (2012). Human organic cation transporter 1 is expressed in lymphoma cells and increases susceptibility to irinotecan and paclitaxel. J Pharmacol Exp Ther.

[25] More SS, Li S, Yee SW, Chen L, Xu Z, Jablons DM, Giacomini KM (2010). Organic cation transporters modulate the uptake and cytotoxicity of picoplatin, a third-generation platinum analogue. Mol Cancer Ther.

[26] Ballestero MR, Monte MJ, Briz O, Jimenez F, Gonzalez-San Martin F, Marin JJ (2006). Expression of transporters potentially involved in the targeting of cytostatic bile acid derivatives to colon cancer and polyps. Biochem Pharmacol.

[27] Nakanishi T, Tamai I (2011). Solute carrier transporters as targets for drug delivery and pharmacological intervention for chemotherapy. J Pharm Sci.

[28] Zhang S, Lovejoy KS, Shima JE, Lagpacan LL, Shu Y, Lapuk A, Chen Y, Komori T, Gray JW, Chen X, Lippard SJ, Giacomini KM (2006). Organic cation transporters are determinants of oxaliplatin cytotoxicity. Cancer Res.

[29] Thomas J, Wang L, Clark RE, Pirmohamed M (2004). Active transport of imatinib into and out of cells: implications for drug resistance. Blood.

[30] Koren-Michowitz M, Buzaglo Z, Ribakovsky E, Schwarz M, Pessach I, Shimoni A, Beider K, Amariglio N, Le-Coutre P, Nagler A (2014). OCT1 genetic variants are associated with long term outcomes in imatinib treated chronic myeloid leukemia patients. Eur J Haematol.

[31] Rumjanek VM, Vidal RS, Maia RC (2013). Multidrug resistance in chronic myeloid leukaemia: how much can we learn from MDR-CML cell lines?. Biosci Rep.

[32] Wang L, Giannoudis A, Austin G, Clark RE (2012). Peroxisome proliferator-activated receptor activation increases imatinib uptake and killing of chronic myeloid leukemia cells. Exp Hematol.

[33] Sacha T, Czekalska S, Foryciarz K, Zawada M, Florek I, Cwynar D, Wator G, Balwierz W, Skotnicki AB (2011). H-oCT1 gene expression as a predictor of major and complete molecular response to imatinib of chronic myeloid leukemia. Single center experience. Przegl Lek.

[34] Jiang X, Zhao Y, Smith C, Gasparetto M, Turhan A, Eaves A, Eaves C (2007). Chronic myeloid leukemia stem cells possess multiple unique features of resistance to BCR-ABL targeted therapies. Leukemia.

[35] Eechoute K, Sparreboom A, Burger H, Franke RM, Schiavon G, Verweij J, Loos WJ, Wiemer EA, Mathijssen RH (2011). Drug transporters and imatinib treatment: implications for clinical practice. Clin Cancer Res.

[36] White DL, Saunders VA, Dang P, Engler J, Venables A, Zrim S, Zannettino A, Lynch K, Manley PW, Hughes T (2007). Most CML patients who have a suboptimal response to imatinib have low OCT-1 activity: higher doses of imatinib may overcome the negative impact of low OCT-1 activity. Blood.

[37] Okuda M, Saito H, Urakami Y, Takano M, Inui K (1996). cDNA cloning and functional expression of a novel rat kidney organic cation transporter, OCT2. Biochem Biophys Res Commun.

[38] Motohashi H, Sakurai Y, Saito H, Masuda S, Urakami Y, Goto M, Fukatsu A, Ogawa O, Inui K (2002). Gene expression levels and immunolocalization of organic ion transporters in the human kidney. J Am Soc Nephrol.

[39] Ciarimboli G, Deuster D, Knief A, Sperling M, Holtkamp M, Edemir B, Pavenstadt H, Lanvers-Kaminsky C, Am Zehnhoff-Dinnesen A, Schinkel AH, Koepsell H, Jurgens H, Schlatter E (2010). Organic cation transporter 2 mediates cisplatin-induced oto- and nephrotoxicity and is a target for protective interventions. Am J Pathol.

[40] Filipski KK, Mathijssen RH, Mikkelsen TS, Schinkel AH, Sparreboom A (2009). Contribution of organic cation transporter 2 (OCT2) to cisplatin-induced nephrotoxicity. Clin Pharmacol Ther.

[41] Franke RM, Kosloske AM, Lancaster CS, Filipski KK, Hu C, Zolk O, Mathijssen RH, Sparreboom A (2010). Influence of Oct1/Oct2-deficiency on cisplatin-induced changes in urinary N-acetyl-beta-D-glucosaminidase. Clin Cancer Res.

[42] Raymond E, Lawrence R, Izbicka E, Faivre S, Von Hoff DD (1998). Activity of oxaliplatin against human tumor colony-forming units. Clin Cancer Res.

[43] Yokoo S, Yonezawa A, Masuda S, Fukatsu A, Katsura T, Inui K (2007). Differential contribution of organic cation transporters, OCT2 and MATE1, in platinum agent-induced nephrotoxicity. Biochem Pharmacol.

[44] Yonezawa A, Masuda S, Yokoo S, Katsura T, Inui K (2006). Cisplatin and oxaliplatin, but not carboplatin and nedaplatin, are substrates for human organic cation transporters (SLC22A1-3 and multidrug and toxin extrusion family). J Pharmacol Exp Ther.

[45] Chen Y, Teranishi K, Li S, Yee SW, Hesselson S, Stryke D, Johns SJ, Ferrin TE, Kwok P, Giacomini KM (2009). Genetic variants in multidrug and toxic compound extrusion-1, hMATE1, alter transport function. Pharmacogenomics J.

[46] Grundemann D, Schechinger B, Rappold GA, Schomig E (1998). Molecular identification of the corticosterone-sensitive extraneuronal catecholamine transporter. Nat Neurosci.

[47] Kekuda R, Prasad PD, Wu X, Wang H, Fei YJ, Leibach FH, Ganapathy V (1998). Cloning and functional characterization of a potential-sensitive, polyspecific organic cation transporter (OCT3) most abundantly expressed in placenta. J Biol Chem.

[48] Nies AT, Koepsell H, Damme K, Schwab M (2011). Organic cation transporters (OCTs, MATEs), in vitro and in vivo evidence for the importance in drug therapy. Handb Exp Pharmacol.

[49] Shang T, Uihlein AV, Van Asten J, Kalyanaraman B, Hillard CJ (2003). 1-Methyl-4-phenylpyridinium accumulates in cerebellar granule neurons via organic cation transporter 3. J Neurochem.

[50] Sata R, Ohtani H, Tsujimoto M, Murakami H, Koyabu N, Nakamura T, Uchiumi T, Kuwano M, Nagata H, Tsukimori K, Nakano H, Sawada Y (2005). Functional analysis of organic cation transporter 3 expressed in human placenta. J Pharmacol Exp Ther.

[51] Yokoo S, Masuda S, Yonezawa A, Terada T, Katsura T, Inui K (2008). Significance of organic cation transporter 3 (SLC22A3) expression for the cytotoxic effect of oxaliplatin in colorectal cancer. Drug Metab Dispos.

[52] Shnitsar V, Eckardt R, Gupta S, Grottker J, Muller GA, Koepsell H, Burckhardt G, Hagos Y (2009). Expression of human organic cation transporter 3 in kidney carcinoma cell lines increases chemosensitivity to melphalan, irinotecan, and vincristine. Cancer Res.

[53] Koepsell H, Endou H (2004). The SLC22 drug transporter family. Pflugers Arch.

[54] Sekine T, Watanabe N, Hosoyamada M, Kanai Y, Endou H (1997). Expression cloning and characterization of a novel multispecific organic anion transporter. J Biol Chem.

[55] Lopez-Nieto CE, You G, Bush KT, Barros EJ, Beier DR, Nigam SK (1997). Molecular cloning and characterization of NKT, a gene product related to the organic cation transporter family that is almost exclusively expressed in the kidney. J Biol Chem.

[56] Reid G, Wolff NA, Dautzenberg FM, Burckhardt G (1998). Cloning of a human renal p-aminohippurate transporter, hROAT1. Kidney Blood Press Res.

[57] Hosoyamada M, Sekine T, Kanai Y, Endou H (1999). Molecular cloning and functional expression of a multispecific organic anion transporter from human kidney. Am J Physiol.

[58] Tahara H, Shono M, Kusuhara H, Kinoshita H, Fuse E, Takadate A, Otagiri M, Sugiyama Y (2005). Molecular cloning and functional analyses of OAT1 and OAT3 from cynomolgus monkey kidney. Pharm Res.

[59] Nomura M, Motohashi H, Sekine H, Katsura T, Inui K (2012). Developmental expression of renal organic anion transporters in rat kidney and its effect on renal secretion of phenolsulfonphthalein. Am J Physiol Renal Physiol.

[60] Hasannejad H, Takeda M, Taki K, Shin HJ, Babu E, Jutabha P, Khamdang S, Aleboyeh M, Onozato ML, Tojo A, Enomoto A, Anzai N, Narikawa S, Huang XL, Niwa T, Endou H (2004). Interactions of human organic anion transporters with diuretics. J Pharmacol Exp Ther.

[61] Uwai Y, Taniguchi R, Motohashi H, Saito H, Okuda M, Inui K (2004). Methotrexate-loxoprofen interaction: involvement of human organic anion transporters hOAT1 and hOAT3. Drug Metab Pharmacokinet.

[62] Nozaki Y, Kusuhara H, Endou H, Sugiyama Y (2004). Quantitative evaluation of the drug-drug interactions between methotrexate and nonsteroidal anti-inflammatory drugs in the renal uptake process based on the contribution of organic anion transporters and reduced folate carrier. J Pharmacol Exp Ther.

[63] Kaler G, Truong DM, Khandelwal A, Nagle M, Eraly SA, Swaan PW, Nigam SK (2007). Structural variation governs substrate specificity for organic anion transporter (OAT) homologs. Potential remote sensing by OAT family members. J Biol Chem.

[64] Mori K, Ogawa Y, Ebihara K, Aoki T, Tamura N, Sugawara A, Kuwahara T, Ozaki S, Mukoyama M, Tashiro K, Tanaka I, Nakao K (1997). Kidney-specific expression of a novel mouse organic cation transporter-like protein. FEBS Lett.

[65] Burckhardt G, Burckhardt BC (2011). In vitro and in vivo evidence of the importance of organic anion transporters (OATs) in drug therapy. Handb Exp Pharmacol.

[66] Fork C, Bauer T, Golz S, Geerts A, Weiland J, Del Turco D, Schomig E, Grundemann D (2011). OAT2 catalyses efflux of glutamate and uptake of orotic acid. Biochem J.

[67] Hilgendorf C, Ahlin G, Seithel A, Artursson P, Ungell AL, Karlsson J (2007). Expression of thirty-six drug transporter genes in human intestine, liver, kidney, and organotypic cell lines. Drug Metab Dispos.

[68] Sun W, Wu RR, van Poelje PD, Erion MD (2001). Isolation of a family of organic anion transporters from human liver and kidney. Biochem Biophys Res Commun.

[69] Cropp CD, Komori T, Shima JE, Urban TJ, Yee SW, More SS, Giacomini KM (2008). Organic anion transporter 2 (SLC22A7) is a facilitative transporter of cGMP. Mol Pharmacol.

[70] Simonson GD, Vincent AC, Roberg KJ, Huang Y, Iwanij V (1994). Molecular cloning and characterization of a novel liver-specific transport protein. J Cell Sci.

[71] Rizwan AN, Burckhardt G (2007). Organic anion transporters of the SLC22 family: biopharmaceutical, physiological, and pathological roles. Pharm Res.

[72] Kobayashi Y, Ohshiro N, Sakai R, Ohbayashi M, Kohyama N, Yamamoto T (2005). Transport mechanism and substrate specificity of human organic anion transporter 2 (hOat2 [SLC22A7]). J Pharm Pharmacol.

[73] Tanino T, Nawa A, Nakao M, Noda M, Fujiwara S, Iwaki M (2009). Organic anion transporting polypeptide 2-mediated uptake of paclitaxel and 2’-ethylcarbonate-linked paclitaxel in freshly isolated rat hepatocytes. J Pharm Pharmacol.

[74] Bleasby K, Castle JC, Roberts CJ, Cheng C, Bailey WJ, Sina JF, Kulkarni AV, Hafey MJ, Evers R, Johnson JM, Ulrich RG, Slatter JG (2006). Expression profiles of 50 xenobiotic transporter genes in humans and pre-clinical species: a resource for investigations into drug disposition. Xenobiotica.

[75] Cha SH, Sekine T, Fukushima JI, Kanai Y, Kobayashi Y, Goya T, Endou H (2001). Identification and characterization of human organic anion transporter 3 expressing predominantly in the kidney. Mol Pharmacol.

[76] Kobayashi Y, Ohshiro N, Tsuchiya A, Kohyama N, Ohbayashi M, Yamamoto T (2004). Renal transport of organic compounds mediated by mouse organic anion transporter 3 (mOat3): further substrate specificity of mOat3. Drug Metab Dispos.

[77] Matsumoto S, Yoshida K, Ishiguro N, Maeda T, Tamai I (2007). Involvement of rat and human organic anion transporter 3 in the renal tubular secretion of topotecan [(S)-9-dimethylaminomethyl-10-hydroxy-camptothecin hydrochloride]. J Pharmacol Exp Ther.

[78] Tamai I, Yabuuchi H, Nezu J, Sai Y, Oku A, Shimane M, Tsuji A (1997). Cloning and characterization of a novel human pH-dependent organic cation transporter, OCTN1. FEBS Lett.

[79] Tamai I, Ohashi R, Nezu J, Yabuuchi H, Oku A, Shimane M, Sai Y, Tsuji A (1998). Molecular and functional identification of sodium ion-dependent, high affinity human carnitine transporter OCTN2. J Biol Chem.

[80] Enomoto A, Wempe MF, Tsuchida H, Shin HJ, Cha SH, Anzai N, Goto A, Sakamoto A, Niwa T, Kanai Y, Anders MW, Endou H (2002). Molecular identification of a novel carnitine transporter specific to human testis. Insights into the mechanism of carnitine recognition. J Biol Chem.

[81] Okabe M, Unno M, Harigae H, Kaku M, Okitsu Y, Sasaki T, Mizoi T, Shiiba K, Takanaga H, Terasaki T, Matsuno S, Sasaki I, Ito S, Abe T (2005). Characterization of the organic cation transporter SLC22A16: a doxorubicin importer. Biochem Biophys Res Commun.

[82] Gong S, Lu X, Xu Y, Swiderski CF, Jordan CT, Moscow JA (2002). Identification of OCT6 as a novel organic cation transporter preferentially expressed in hematopoietic cells and leukemias. Exp Hematol.

[83] Jong NN, Nakanishi T, Liu JJ, Tamai I, McKeage MJ (2011). Oxaliplatin transport mediated by organic cation/carnitine transporters OCTN1 and OCTN2 in overexpressing human embryonic kidney 293 cells and rat dorsal root ganglion neurons. J Pharmacol Exp Ther.

[84] Hu S, Franke RM, Filipski KK, Hu C, Orwick SJ, de Bruijn EA, Burger H, Baker SD, Sparreboom A (2008). Interaction of imatinib with human organic ion carriers. Clin Cancer Res.

[85] Peng B, Lloyd P, Schran H (2005). Clinical pharmacokinetics of imatinib. Clin Pharmacokinet.

[86] Angelini S, Pantaleo MA, Ravegnini G, Zenesini C, Cavrini G, Nannini M, Fumagalli E, Palassini E, Saponara M, Di Battista M, Casali PG, Hrelia P, Cantelli-Forti G, Biasco G (2013). Polymorphisms in OCTN1 and OCTN2 transporters genes are associated with prolonged time to progression in unresectable gastrointestinal stromal tumours treated with imatinib therapy. Pharmacol Res.

[87] Aouida M, Poulin R, Ramotar D (2010). The human carnitine transporter SLC22A16 mediates high affinity uptake of the anticancer polyamine analogue bleomycin-A5. J Biol Chem.

[88] Meier PJ, Eckhardt U, Schroeder A, Hagenbuch B, Stieger B (1997). Substrate specificity of sinusoidal bile acid and organic anion uptake systems in rat and human liver. Hepatology.

[89] Hagenbuch B, Meier PJ (2004). Organic anion transporting polypeptides of the OATP/SLC21 family: phylogenetic classification as OATP/SLCO superfamily, new nomenclature and molecular/functional properties. Pflugers Arch.

[90] Hagenbuch B, Meier PJ (2003). The superfamily of organic anion transporting polypeptides. Biochim Biophys Acta.

[91] Hagenbuch B, Gui C (2008). Xenobiotic transporters of the human organic anion transporting polypeptides (OATP) family. Xenobiotica.

[92] Kullak-Ublick GA, Beuers U, Paumgartner G (1996). Molecular and functional characterization of bile acid transport in human hepatoblastoma HepG2 cells. Hepatology.

[93] Abe T, Kakyo M, Tokui T, Nakagomi R, Nishio T, Nakai D, Nomura H, Unno M, Suzuki M, Naitoh T, Matsuno S, Yawo H (1999). Identification of a novel gene family encoding human liver-specific organic anion transporter LST-1. J Biol Chem.

[94] Hsiang B, Zhu Y, Wang Z, Wu Y, Sasseville V, Yang WP, Kirchgessner TG (1999). A novel human hepatic organic anion transporting polypeptide (OATP2). Identification of a liver-specific human organic anion transporting polypeptide and identification of rat and human hydroxymethylglutaryl-CoA reductase inhibitor transporters. J Biol Chem.

[95] Konig J, Cui Y, Nies AT, Keppler D (2000). A novel human organic anion transporting polypeptide localized to the basolateral hepatocyte membrane. Am J Physiol Gastrointest Liver Physiol.

[96] Okabe M, Szakacs G, Reimers MA, Suzuki T, Hall MD, Abe T, Weinstein JN, Gottesman MM (2008). Profiling SLCO and SLC22 genes in the NCI-60 cancer cell lines to identify drug uptake transporters. Mol Cancer Ther.

[97] Nozawa T, Minami H, Sugiura S, Tsuji A, Tamai I (2005). Role of organic anion transporter OATP1B1 (OATP-C) in hepatic uptake of irinotecan and its active metabolite, 7-ethyl-10-hydroxycamptothecin: in vitro evidence and effect of single nucleotide polymorphisms. Drug Metab Dispos.

[98] Takane H, Kawamoto K, Sasaki T, Moriki K, Kitano H, Higuchi S, Otsubo K, Ieiri I (2009). Life-threatening toxicities in a patient with UGT1A1*6/*28 and SLCO1B1*15/*15 genotypes after irinotecan-based chemotherapy. Cancer Chemother Pharmacol.

[99] van de Steeg E, van der Kruijssen CM, Wagenaar E, Burggraaff JE, Mesman E, Kenworthy KE, Schinkel AH (2009). Methotrexate pharmacokinetics in transgenic mice with liver-specific expression of human organic anion-transporting polypeptide 1B1 (SLCO1B1). Drug Metab Dispos.

[100] Ni W, Ji J, Dai Z, Papp A, Johnson AJ, Ahn S, Farley KL, Lin TS, Dalton JT, Li X, Jarjoura D, Byrd JC, Sadee W, Grever MR, Phelps MA (2010). Flavopiridol pharmacogenetics: clinical and functional evidence for the role of SLCO1B1/OATP1B1 in flavopiridol disposition. PLoS One.

[101] Katz DA, Carr R, Grimm DR, Xiong H, Holley-Shanks R, Mueller T, Leake B, Wang Q, Han L, Wang PG, Edeki T, Sahelijo L, Doan T, Allen A, Spear BB (2006). Organic anion transporting polypeptide 1B1 activity classified by SLCO1B1 genotype influences atrasentan pharmacokinetics. Clin Pharmacol Ther.

[102] Liu T, Li Q (2014). Organic anion-transporting polypeptides: a novel approach for cancer therapy. J Drug Target.

[103] Lee W, Belkhiri A, Lockhart AC, Merchant N, Glaeser H, Harris EI, Washington MK, Brunt EM, Zaika A, Kim RB, El-Rifai W (2008). Overexpression of OATP1B3 confers apoptotic resistance in colon cancer. Cancer Res.

[104] Lockhart AC, Harris E, Lafleur BJ, Merchant NB, Washington MK, Resnick MB, Yeatman TJ, Lee W (2008). Organic anion transporting polypeptide 1B3 (OATP1B3) is overexpressed in colorectal tumors and is a predictor of clinical outcome. Clin Exp Gastroenterol.

[105] Wright JL, Kwon EM, Ostrander EA, Montgomery RB, Lin DW, Vessella R, Stanford JL, Mostaghel EA (2011). Expression of SLCO transport genes in castration-resistant prostate cancer and impact of genetic variation in SLCO1B3 and SLCO2B1 on prostate cancer outcomes. Cancer Epidemiol Biomarkers Prev.

[106] Abe T, Unno M, Onogawa T, Tokui T, Kondo TN, Nakagomi R, Adachi H, Fujiwara K, Okabe M, Suzuki T, Nunoki K, Sato E, Kakyo M, Nishio T, Sugita J, Asano N, Tanemoto M, Seki M, Oate F, Ono K, Kondo Y, Shiibak K, Suzuki M, Ohtani H, Shimosegawa T, Iinuma K, Nagura H, Ito S, Matsuno S (2001). LST-2, a human liver-specific organic anion transporter, determines methotrexate sensitivity in gastrointestinal cancers. Gastroenterology.

[107] Smith NF, Acharya MR, Desai N, Figg WD, Sparreboom A (2005). Identification of OATP1B3 as a high-affinity hepatocellular transporter of paclitaxel. Cancer Biol Ther.

[108] Garcia AD, Ostapchuk P, Hearing P (1991). Methylation-dependent and -independent DNA binding of nuclear factor EF-C. Virology.

[109] Yamaguchi H, Kobayashi M, Okada M, Takeuchi T, Unno M, Abe T, Goto J, Hishinuma T, Mano N (2008). Rapid screening of antineoplastic candidates for the human organic anion transporter OATP1B3 substrates using fluorescent probes. Cancer Lett.

[110] Maekawa M, Tanaka H (1991). [Hemofiltration and computer]. Nihon Rinsho.

[111] Glaeser H, Bailey DG, Dresser GK, Gregor JC, Schwarz UI, McGrath JS, Jolicoeur E, Lee W, Leake BF, Tirona RG, Kim RB (2007). Intestinal drug transporter expression and the impact of grapefruit juice in humans. Clin Pharmacol Ther.

[112] Gao B, Hagenbuch B, Kullak-Ublick GA, Benke D, Aguzzi A, Meier PJ (2000). Organic anion-transporting polypeptides mediate transport of opioid peptides across blood-brain barrier. J Pharmacol Exp Ther.

[113] Liedauer R, Svoboda M, Wlcek K, Arrich F, Ja W, Toma C, Thalhammer T (2009). Different expression patterns of organic anion transporting polypeptides in osteosarcomas, bone metastases and aneurysmal bone cysts. Oncol Rep.

[114] van de Steeg E, van Esch A, Wagenaar E, Kenworthy KE, Schinkel AH (2013). Influence of human OATP1B1, OATP1B3, and OATP1A2 on the pharmacokinetics of methotrexate and paclitaxel in humanized transgenic mice. Clin Cancer Res.

[115] van de Steeg E, van Esch A, Wagenaar E, van der Kruijssen CM, van Tellingen O, Kenworthy KE, Schinkel AH (2011). High impact of Oatp1a/1b transporters on in vivo disposition of the hydrophobic anticancer drug paclitaxel. Clin Cancer Res.

[116] Wlcek K, Svoboda M, Riha J, Zakaria S, Olszewski U, Dvorak Z, Sellner F, Ellinger I, Jager W, Thalhammer T (2011). The analysis of organic anion transporting polypeptide (OATP) mRNA and protein patterns in primary and metastatic liver cancer. Cancer Biol Ther.

[117] Kim H, Son HY, Bailey SM, Lee J (2009). Deletion of hepatic Ctr1 reveals its function in copper acquisition and compensatory mechanisms for copper homeostasis. Am J Physiol Gastrointest Liver Physiol.

[118] Zhou B, Gitschier J (1997). hCTR1: a human gene for copper uptake identified by complementation in yeast. Proc Natl Acad Sci U S A.

[119] Lee J, Pena MM, Nose Y, Thiele DJ (2002). Biochemical characterization of the human copper transporter Ctr1. J Biol Chem.

[120] Holzer AK, Varki NM, Le QT, Gibson MA, Naredi P, Howell SB (2006). Expression of the human copper influx transporter 1 in normal and malignant human tissues. J Histochem Cytochem.

[121] Ishida S, Lee J, Thiele DJ, Herskowitz I (2002). Uptake of the anticancer drug cisplatin mediated by the copper transporter Ctr1 in yeast and mammals. Proc Natl Acad Sci U S A.

[122] Howell SB, Safaei R, Larson CA, Sailor MJ (2010). Copper transporters and the cellular pharmacology of the platinum-containing cancer drugs. Mol Pharmacol.

[123] Kuo MT, Chen HH, Song IS, Savaraj N, Ishikawa T (2007). The roles of copper transporters in cisplatin resistance. Cancer Metastasis Rev.

[124] Song IS, Savaraj N, Siddik ZH, Liu P, Wei Y, Wu CJ, Kuo MT (2004). Role of human copper transporter Ctr1 in the transport of platinum-based antitumor agents in cisplatin-sensitive and cisplatin-resistant cells. Mol Cancer Ther.

[125] Liang ZD, Stockton D, Savaraj N, Tien Kuo M (2009). Mechanistic comparison of human high-affinity copper transporter 1-mediated transport between copper ion and cisplatin. Mol Pharmacol.

[126] Pabla N, Murphy RF, Liu K, Dong Z (2009). The copper transporter Ctr1 contributes to cisplatin uptake by renal tubular cells during cisplatin nephrotoxicity. Am J Physiol Renal Physiol.

[127] Morita Y, Kodama K, Shiota S, Mine T, Kataoka A, Mizushima T, Tsuchiya T (1998). NorM, a putative multidrug efflux protein, of Vibrio parahaemolyticus and its homolog in Escherichia coli. Antimicrob Agents Chemother.

[128] Otsuka M, Matsumoto T, Morimoto R, Arioka S, Omote H, Moriyama Y (2005). A human transporter protein that mediates the final excretion step for toxic organic cations. Proc Natl Acad Sci U S A.

[129] Yonezawa A, Inui K (2011). Importance of the multidrug and toxin extrusion MATE/SLC47A family to pharmacokinetics, pharmacodynamics/toxicodynamics and pharmacogenomics. Br J Pharmacol.

[130] Ahmadimoghaddam D, Zemankova L, Nachtigal P, Dolezelova E, Neumanova Z, Cerveny L, Ceckova M, Kacerovsky M, Micuda S, Staud F (2013). Organic cation transporter 3 (OCT3/SLC22A3) and multidrug and toxin extrusion 1 (MATE1/SLC47A1) transporter in the placenta and fetal tissues: expression profile and fetus protective role at different stages of gestation. Biol Reprod.

[131] Komatsu T, Hiasa M, Miyaji T, Kanamoto T, Matsumoto T, Otsuka M, Moriyama Y, Omote H (2011). Characterization of the human MATE2 proton-coupled polyspecific organic cation exporter. Int J Biochem Cell Biol.

[132] Nakamura T, Yonezawa A, Hashimoto S, Katsura T, Inui K (2010). Disruption of multidrug and toxin extrusion MATE1 potentiates cisplatin-induced nephrotoxicity. Biochem Pharmacol.

[133] Li Q, Peng X, Yang H, Wang H, Shu Y (2011). Deficiency of multidrug and toxin extrusion 1 enhances renal accumulation of paraquat and deteriorates kidney injury in mice. Mol Pharm.

[134] Yonezawa A (2012). [Platinum agent-induced nephrotoxicity via organic cation transport system]. Yakugaku Zasshi.

[135] Rubio-Aliaga I, Daniel H (2008). Peptide transporters and their roles in physiological processes and drug disposition. Xenobiotica.

[136] Terada T, Inui K (2012). Recent advances in structural biology of peptide transporters. Curr Top Membr.

[137] Nakanishi T, Tamai I, Sai Y, Sasaki T, Tsuji A (1997). Carrier-mediated transport of oligopeptides in the human fibrosarcoma cell line HT1080. Cancer Res.

[138] Gonzalez DE, Covitz KM, Sadee W, Mrsny RJ (1998). An oligopeptide transporter is expressed at high levels in the pancreatic carcinoma cell lines AsPc-1 and Capan-2. Cancer Res.

[139] Anderson CM, Jevons M, Thangaraju M, Edwards N, Conlon NJ, Woods S, Ganapathy V, Thwaites DT (2010). Transport of the photodynamic therapy agent 5-aminolevulinic acid by distinct H + -coupled nutrient carriers coexpressed in the small intestine. J Pharmacol Exp Ther.

[140] Nakanishi T, Tamai I, Takaki A, Tsuji A (2000). Cancer cell-targeted drug delivery utilizing oligopeptide transport activity. Int J Cancer.

[141] Mitsuoka K, Kato Y, Miyoshi S, Murakami Y, Hiraiwa M, Kubo Y, Nishimura S, Tsuji A (2010). Inhibition of oligopeptide transporter suppress growth of human pancreatic cancer cells. Eur J Pharm Sci.

[142] Grem JL (2000). 5-Fluorouracil: forty-plus and still ticking. A review of its preclinical and clinical development. Invest New Drugs.

[143] Kawaguchi T, Saito M, Suzuki Y, Nambu N, Nagai T (1985). Specificity of esterases and structure of prodrug esters. II. Hydrolytic regeneration behavior of 5-fluoro-2’-deoxyuridine (FUdR) from 3’,5’-diesters of FUdR with rat tissue homogenates and plasma in relation to their antitumor activity. Chem Pharm Bull (Tokyo).

[144] Vig BS, Lorenzi PJ, Mittal S, Landowski CP, Shin HC, Mosberg HI, Hilfinger JM, Amidon GL (2003). Amino acid ester prodrugs of floxuridine: synthesis and effects of structure, stereochemistry, and site of esterification on the rate of hydrolysis. Pharm Res.

[145] Han HK, Oh DM, Amidon GL (1998). Cellular uptake mechanism of amino acid ester prodrugs in Caco-2/hPEPT1 cells overexpressing a human peptide transporter. Pharm Res.

[146] Landowski CP, Vig BS, Song X, Amidon GL (2005). Targeted delivery to PEPT1-overexpressing cells: acidic, basic, and secondary floxuridine amino acid ester prodrugs. Mol Cancer Ther.

[147] Taylor PM (2014). Role of amino acid transporters in amino acid sensing. Am J Clin Nutr.

[148] Bjornsti MA, Houghton PJ (2004). The TOR pathway: a target for cancer therapy. Nat Rev Cancer.

[149] Yang Q, Guan KL (2007). Expanding mTOR signaling. Cell Res.

[150] Nicklin P, Bergman P, Zhang B, Triantafellow E, Wang H, Nyfeler B, Yang H, Hild M, Kung C, Wilson C, Myer VE, Mackeigan JP, Porter JA, Wang YK, Cantley LC, Finan PM, Murphy LO (2009). Bidirectional transport of amino acids regulates mTOR and autophagy. Cell.

[151] Kondoh N, Imazeki N, Arai M, Hada A, Hatsuse K, Matsuo H, Matsubara O, Ohkura S, Yamamoto M (2007). Activation of a system A amino acid transporter, ATA1/SLC38A1, in human hepatocellular carcinoma and preneoplastic liver tissues. Int J Oncol.

[152] O’Dwyer PJ, Alonso MT, Leyland-Jones B (1984). Acivicin: a new glutamine antagonist in clinical trials. J Clin Oncol.

[153] Geier EG, Schlessinger A, Fan H, Gable JE, Irwin JJ, Sali A, Giacomini KM (2013). Structure-based ligand discovery for the Large-neutral Amino Acid Transporter 1, LAT-1. Proc Natl Acad Sci U S A.

[154] Patel M, Dalvi P, Gokulgandhi M, Kesh S, Kohli T, Pal D, Mitra AK (2013). Functional characterization and molecular expression of large neutral amino acid transporter (LAT1) in human prostate cancer cells. Int J Pharm.

